# Synthesis and evaluation of [^11^C]MMPIP as a potential radioligand for imaging of metabotropic glutamate 7 receptor in the brain

**DOI:** 10.1186/2191-219X-3-54

**Published:** 2013-07-22

**Authors:** Tomoteru Yamasaki, Katsushi Kumata, Joji Yui, Masayuki Fujinaga, Kenji Furutsuka, Akiko Hatori, Lin Xie, Masanao Ogawa, Nobuki Nengaki, Kazunori Kawamura, Ming-Rong Zhang

**Affiliations:** 1 Molecular Probe Program, Molecular Imaging Center, National Institute of Radiological Sciences, 4-9-1 Anagawa, Inage-ku, Chiba 263-8555, Japan; 2 SHI Accelerator Service Co. Ltd., 5-9-11 Kitashinagawa, Shinagawa-ku, Tokyo 141-8686, Japan

**Keywords:** MMPIP, mGlu7, PET, ^11^C, Autoradiography, Specific activity

## Abstract

**Background:**

Metabotropic glutamate 7 (mGlu7) receptor is a crucial target protein for the development of pharmaceuticals against central nervous system disorders. In the present study, we synthesized [^11^C]MMPIP, a putative radioligand for mGlu7 (binding constant *K*_B_ = 30 nM), and evaluated its potential for imaging of mGlu7 via *in vitro* and *in vivo* techniques.

**Methods:**

[^11^C]MMPIP was synthesized by the reaction of phenol precursor **3** with [^11^C]CH_3_I. *In vitro* autoradiography using [^11^C]MMPIP was performed on rat brain sections. To determine *in vitro* specific binding of [^11^C]MMPIP with mGlu7, a blocking study was conducted by co-incubation with excess AMN082, a selective antagonist for mGlu7, or unlabeled MMPIP. Positron emission tomography (PET) studies and *ex vivo* metabolite analysis were carried out on rat brains.

**Results:**

[^11^C]MMPIP was obtained with two specific activity (SA) levels of average 58 (conventional) and 3,800 (high SA) GBq/μmol, respectively. High radioactive signals derived from conventional [^11^C]MMPIP in the *in vitro* autoradiography were seen in the thalamus, medulla oblongata, and striatum, corresponding with comprehensive brain distributions of mGlu7. Co-incubation with ANM082 or unlabeled MMPIP reduced the radioactive signals in the brain sections, respectively. In the PET studies with [^11^C]MMPIP, no specific uptake relative to mGlu7 was found in the examined brain regions.

**Conclusion:**

Despite *in vitro* specific binding of [^11^C]MMPIP with mGlu7, visualization of mGlu7 in the living brain using PET was not successful. Development of new ligand candidates with higher affinity for mGlu7 is necessary.

## Background

Glutamate is a dominant neurotransmitter in the mammalian central nervous system (CNS), inducing excitatory neurotransmission by binding to its receptors. Glutamate receptors are classified as metabotropic or iontropic types based on their biological functions and molecular structures [1,2]. Eight subtypes of metabotropic glutamate receptors (mGlu1 to 8) have been identified and divided into three groups based on sequence homology, pharmacology, and coupling pathway to G proteins [3]. So far, major drug discovery programs have largely focused on group I (mGlu1 and mGlu5) and II (mGlu2 and mGlu3), which have been implicated in various neuropathological and psychiatric disorders [4-6]. On the other hand, the group III receptors including four subtypes, such as mGlu4 and mGlu6 to 8, are less understood, mainly due to the paucity of specific ligands for these receptors.

Of the group III receptors, mGlu7 is mainly localized on the presynaptic terminals of glutamatergic neurons, and its signaling pathways are activated only under conditions of emergency synaptic activity because of its low affinity to glutamate [7]. Studies on mGlu7-deficient mice have directly demonstrated that mGlu7 plays the most important role in controlling neuronal excitability via feedback regulation of glutamate in the synaptic cleft [8]. Moreover, mGlu7 is known to regulate glutamate release through modulation of cAMP levels in cerebrocortical nerve terminals [9]. Recent advances in the generation of transgenic animals and the identification of selective or specific ligands have revealed important insights into the potential role of mGlu7 in the pathophysiology of neurological disorders [10,11].

**Figure 1:**
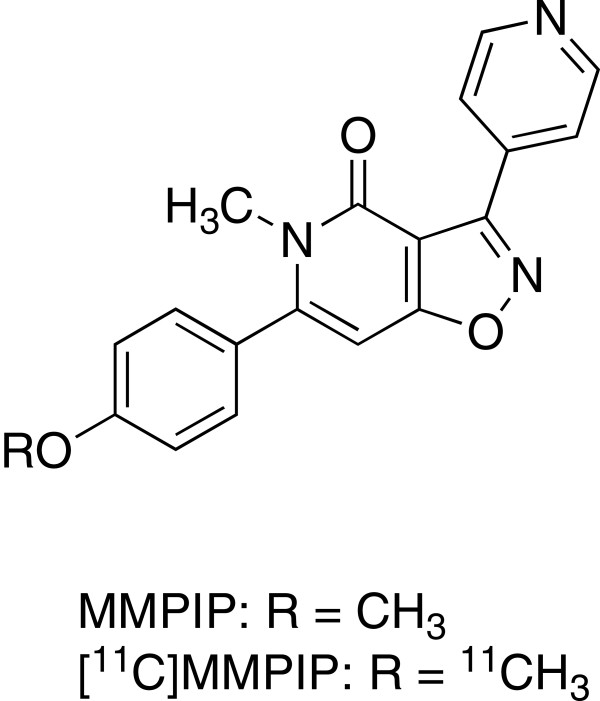
Chemical structures.

MMPIP     (6-(4-methoxyphenyl-5-methyl-3-pyridin-4-ylisozazolo[4,5-*c*]pyridine-4(5*H*)-one, Figure 1) has been previously developed as an allosteric antagonist for mGlu7 [11]. MMPIP intercepts agonist-induced Gq-coupled Ca^2+^ mobilization and the Gi-coupled cAMP pathway and is used as a promising pharmacological tool for elucidating the role of mGlu7 in CNS functions [12,13]. The binding constant (*K*_B_) value of MMPIP against binding of l-(+)-2-amino-4-phosphonobutyric acid (l-AP4), an agonist for mGlu7-induced Ca^2+^ mobilization, with mGlu7 was 30 nM. In addition, IC_50_ values of MMPIP against l-AP4-induced cAMP accumulation in Chinese hamster ovary (CHO)-rat mGlu7 and CHO-human mGlu7 cells were 220 ± 23 nM and 610 ± 130 nM, respectively [12].

Positron emission tomography (PET) is frequently used as a tool for basic and clinical studies to elucidate the distribution, function, and downregulation or upregulation of neuroreceptors in living brains of animals and human. Although mGlu7 is known to be an important research target receptor, to the best of our knowledge, there have been no PET ligands developed for the imaging of mGlu7 until now. In this study, we aimed to develop ^11^C-labeled MMPIP ([^11^C]MMPIP, Figure 1) and to evaluate its potential as the first putative radioligand for imaging of mGlu7 in the brain. Here, we describe the following:

1. Radiosynthesis of [^11^C]MMPIP with conventional (average 58 GBq/μmol) and high specific activity (SA) (average 3,800 GBq/μmol)

2. *In vitro* autoradiography using rat brain sections

3. PET studies with [^11^C]MMPIP of two SA levels on rat brains

4. *Ex vivo* metabolite analysis in the plasma and brain of rats

## Methods

### Materials

The melting point (mp) was measured using a micro-melting point apparatus (MP-500P, Yanaco, Tokyo, Japan) and uncorrected. ^1^H-NMR (300 MHz) spectra were recorded on a spectrometer (JEOL-AL-300, JEOL, Tokyo, Japan) with tetramethylsilane as an internal standard. All chemical shifts (*δ*) were reported in parts per million downfield relative to the chemical shift of tetramethylsilane. High-resolution mass spectra (HRMS) were obtained on a JEOL NMS-SX102 spectrometer (JEOL). All chemicals and solvents were of analytic or high-performance liquid chromatography (HPLC) grade and were purchased from two commercial suppliers (Sigma-Aldrich, St. Louis, MO, USA and Wako Pure Industries, Osaka, Japan). Column chromatography was performed using Wako gel C-200 (70 to 230 mesh). The authentic MMPIP was prepared according to procedures reported previously [11,14]. The selective mGlu7 antagonist AMN082 was purchased from TOCRIS Bioscience (Bristol, UK).

### Chemical synthesis

#### 5-(2-(4-tert-Butyl-dimethylsilyloxyphenyl)-2-oxoethyl)-N-methyl-3-(pyridin-4-yl)isoxazole-4-carboxamide (1)

A solution of lithium bis(trimethysilyl)amide in hexane (9.6 mL, 1.0 M) was added dropwise to a solution of *N*-(5-methyl)-3-(pyridin-4-yl)isoxazole-4-carboxamide [11,14] (0.87 g, 4.0 mmol) in anhydrous tetrahydrofuran (THF, 20 mL) under N_2_ gas at −40°C. The reaction mixture was continuously stirred at −40°C for 30 min. A solution of methyl 4-*tert*-butyl-dimethylsilyloxybenzoate (2.66 g, 10 mmol) in anhydrous THF (20 mL) was added dropwise to the mixture at −40°C. The reaction mixture was stirred at room temperature overnight. The mixture was treated with a saturated NH_4_Cl solution and extracted with ethyl acetate. After the organic layer was washed with a saturated NaCl solution, dried with MgSO_4_, and evaporated under reduced pressure, the crude product was purified by column chromatography with CH_2_Cl_2_/CH_3_OH (99:1) to give **1** (1.31 g, 72.6%) as an amorphous compound. ^1^H-NMR (CDCl_3_, *δ*): 8.74 (2H, dd, *J* = 2.1, 4.6 Hz), 8.02 (2H, d, *J* = 8.8 Hz), 7.71 (2H, dd, *J* = 2.1, 4.4 Hz), 7.17 (1H, br), 6.96 (2H, dd, *J* = 2.9, 6.8 Hz), 4.62 (2H, s), 2.92 (3H, d, *J* = 12.1 Hz), 1.00 (9H, s), 0.27 (6H, s). HRMS *m*/*z* 451.1924 (calculated for C_24_H_29_N_3_O_4_Si: 451.1927).

#### 5-(2-(4-Hydroxyphenyl)-2-oxoethyl)-N-methyl-3-(pyridin-4-yl)isoxazole-4-carboxamide (2)

To a solution of **1** (1.26 g, 2.8 mmol) in anhydrous THF (5 mL), a solution of tetrabutylammonium fluoride in THF (5 mL, 1.0 M) was added. This reaction mixture was stirred at room temperature overnight. The mixture was treated with water and extracted with ethyl acetate. After the organic layer was washed with a saturated NaCl solution, dried with MgSO_4_, and evaporated under reduced pressure, the crude product was purified by column chromatography with CH_2_Cl_2_/MeOH (99:1) to give **2** (0.59 g, 62.0%) as a colorless solid; mp: 194°C to 196°C. ^1^H-NMR (DMSO-*d*_6_, *δ*): 10.55 (1H, s), 8.73 (2H, dd, *J* = 1.5, 4.8 Hz), 8.22 (1H, d, *J* = 4.8 Hz), 7.93 (2H, d, *J* = 8.8 Hz), 7.65 (2H, dd, *J* = 1.5, 4.8 Hz), 6.91 (2H, d, *J* = 8.4 Hz), 4.80 (2H, s), 2.66 (3H, d, *J* = 4.4 Hz). HRMS *m*/*z* 337.1060 (calculated for C_18_H_15_N_3_O_4_: 337.1063).

#### 6-(4-Hydroxyphenyl)-5-methyl-3-(pyridin-4-yl)isoxazolo[4,5-c]pyridin-4(5H)-one (3)

*p*-Toluenesulfonic acid monohydrate (310 mg, 1.6 mmol) was added to a suspension of **2** (550 mg, 1.6 mmol) in 1,4-dioxane (60 mL). The reaction mixture was heated at reflux for 12 h. This mixture was treated with a saturated Na_2_CO_3_ solution and extracted with ethyl acetate. The organic layer was washed with a saturated NaCl solution, dried with MgSO_4_, and evaporated under reduced pressure. The crude product was then purified by column chromatography with CH_2_Cl_2_ in CH_3_OH (1% to 5%) to give **3** (20.0 mg, 3.8%; overall yield: 1.7% from **1**) as a colorless solid; mp: 189°C. ^1^H-NMR (DMSO-*d*_6_, *δ*): 10.00 (1H, s), 8.80 (2H, dd, *J* = 1.6, 4.6 Hz), 8.24 (2H, dd, *J* = 1.7, 4.6 Hz), 7.39 (2H, d, *J* = 8.4 Hz), 6.92 (2H, d, *J* = 8.8 Hz), 6.83 (2H, s), 3.34 (3H, s). HRMS *m*/*z* 319.0954 (calculated for C_18_H_13_N_3_O_3_: 319.0957).

### Radiosynthesis of [^11^C]MMPIP

#### Conventional SA

[^11^C]CH_3_I was synthesized from cyclotron-produced [^11^C]CO_2_ as described previously [15]. Briefly, [^11^C]CO_2_ was bubbled into 50 μM LiAlH_4_ in anhydrous THF (0.5 mL). After evaporation of THF, the remaining complexes were treated with 57% HI (0.3 mL) to produce [^11^C]CH_3_I, which was transferred under N_2_ gas with heating into a reaction vessel containing anhydrous DMF (0.3 mL), **3** (1.0 mg), and NaOH (0.01 mL, 0.5 M) cooled to −15°C to −20°C. After the radioactivity reached a plateau, the reaction vessel was warmed to 80°C. After 3 min of reaction at 80°C, CH_3_CN/H_2_O (55:45, 0.5 mL) was added to terminate the reaction. The radioactive mixture was applied to a semi-preparative HPLC system. HPLC purification was completed on a Capcell Pak UG-80 C_18_ column (10 mm ID × 250 mm; Shiseido, Tokyo, Japan) using a mobile phase of CH_3_CN/H_2_O (55:45) at a flow rate of 5.0 mL/min. The retention time (*t*_R_) for [^11^C]MMPIP was 9.5 min, whereas for **3**, 6.7 min. The radioactive fraction corresponding to [^11^C]MMPIP was collected in a sterile flask containing polysorbate 80 (75 μL) and ethanol (150 μL), evaporated to dryness under vacuum, re-dissolved in sterile normal saline (3.0 mL), and passed through a 0.22-μm Millipore filter (Billerica, MA, USA) for analysis and animal experiments.

The radiochemical purity of [^11^C]MMPIP was assayed by analytical HPLC (Capcell Pak UG-80 C_18_, 4.6 mm ID × 250 mm; CH_3_CN/H_2_O = 55:45, 1.0 mL/min) coupled with a UV detector (UV-2075 plus, JASCO, Tokyo, Japan). The identity of [^11^C]MMPIP was confirmed by co-injection with the unlabeled MMPIP. The SA of [^11^C]MMPIP was calculated by a comparison of the assayed radioactivity to the carrier mass which was measured from its UV peak at 254 nm.

To determine the stability of [^11^C]MMPIP with conventional SA, after this product was maintained for 120 min at room temperature, analytic sample was taken from the formulated solution of [^11^C]MMPIP to measure the radiochemical purity in the solution.

#### High SA

[^11^C]CH_3_I for radiosynthesis was synthesized by the single-pass I_2_ method as described previously [16]. The cyclotron-produced [^11^C]CH_4_ was passed through a heated I_2_ column and converted to [^11^C]CH_3_I, which was then reacted with **3** (1.0 mg) in the presence of NaOH (10 μL, 0.5 M). After the reaction, the radioactive mixture was treated as described for the conventional SA.

The product was analyzed to measure SA and confirm the identity by radio-HPLC (Waters XBridge Shield RP18, 2.5 μm, 3.0 × 50 mm) coupled with a UV detector (Waters 2487, 210 nm). The mobile phase was a mixture of CH_3_CN and 100 mM ammonium phosphate buffer (pH 2.0) containing 5 mM sodium 1-octanesulfonate with a ratio of 40:60 under a flow rate of 1.0 mL/min. A solution of unlabeled MMPIP at a concentration of 19.35, 25.8, 64.5, 129, and 258 pmol/mL was prepared from a standard solution (2.58 μmol/mL CH_3_CN). A calibration curve was obtained by measuring these solutions three times at each measurement. The high SA product was loaded into a sample loop (5 μL) and injected into the HPLC. The SA value was decay-corrected to the time when the radioactivity of product was measured at the end of synthesis (EOS). The detection limit of this technique was 15.3 pmol/mL under this analytic condition.

To determine the stability of [^11^C]MMPIP with high SA, after this product was maintained for 120 min at room temperature, analytic sample was taken from the formulated solution of [^11^C]MMPIP to measure the radiochemical purity in the solution.

### Measurement and computation of partition coefficient

Partition coefficient (Log *D*) values were measured by mixing conventional SA [^11^C]MMPIP (radiochemical purity 99.3%, about 160,000 cpm) with *n*-octanol (3.0 g) and sodium phosphate buffer saline (PBS, 3.0 g; 0.1 M, pH 7.40) in a test tube. The tube was vortexed for 3 min at room temperature, followed by centrifugation with AT-2724 rotor (KUBOTA, Tokyo, Japan) at 3,500 rpm for 5 min. An aliquot of 1 mL PBS and 1 mL *n*-octanol was removed, weighted, and counted, respectively. Samples from the remaining organic layer were removed and re-partitioned until consistent Log *D* value was obtained. The Log *D* value was calculated by comparing the ratio of counts per minute per gram of *n*-octanol to that of PBS and expressed as Log *D* = Log[cpm/g (*n*-octanol)/cpm/g (PBS)]. All assays were performed in triplicate. Meanwhile, the value of cLog *D* of [^11^C]MMPIP was computed using Pallas 3.4 software (CompuDrug, Sedona, AZ, USA).

### Animals

Sprague–Dawley (SD) rats were purchased from Japan SLC (Shizuoka, Japan). The animal experimental procedures were approved by the Animal Ethics Committee of the National Institute of Radiological Sciences (Chiba, Japan).

### *In vitro* autoradiography

Sagittal sections (8 μm) were prepared from a frozen rat brain using a cryostat (HM560, Carl Zeiss, Oberkochen, Germany). Brain sections were pre-incubated in a 50 mM Trizma buffer containing 1.2 mM MgCl_2_ and 2.0 mM CaCl_2_ at room temperature for 20 min. After [^11^C]MMPIP (74 MBq, 0.7 nmol) was added to the incubation buffer (0.2 L), the brain sections were incubated in the buffer for 60 min at room temperature. To determine the specific binding of [^11^C]MMPIP for mGlu7, we added AMN082, a selective antagonist for mGlu7 [17], or unlabeled MMPIP (10 μM, dissolved in saline containing 20% ethanol and 10% polysorbate 80) to each incubation buffer in advance, respectively. After incubation, these sections were washed three times for 5 min each time with cold buffer, dipped in cold distilled water, and dried with cold air. These sections were placed in contact with an imaging plate (BAS-MS2025, Fujifilm, Tokyo, Japan). Autoradiograms were acquired using a Bio-Imaging Analyzer System (BAS5000, Fujifilm, Tokyo, Japan). The regions of interest (ROIs) were manually drawn on the cerebral cortex, striatum, hippocampus, thalamus, cerebellum, middle peduncle, and medulla oblongata. The radioactivity in the ROIs was quantified and expressed as photo-stimulated luminescence per unit area (PSL/mm^2^).

### Small-animal PET

A male SD rat (*n* = 3, 280 to 385 g; 7 to 10 weeks old) was secured in a custom-designed chamber and placed in a small-animal PET scanner (Inveon, Siemens Medical Solutions, Knoxville, TN, USA). A 24-gauge intravenous catheter (Terumo Medical Products, Somerset, NJ, USA) was placed in the tail vein for a bolus injection. The rat was kept under anesthesia with 1.5% (*v*/*v*) isoflurane during the scan, and its body temperature was maintained with a 40°C water circulation system (T/Pump TP401, Gaymar Industries, Orchard Park, NY, USA).

The dynamic emission scans in 3D list mode were performed for 60 min (1 min × 4 frames, 2 min × 8 frames, and 5 min × 8 frames) on a rat brain after intravenous injection of [^11^C]MMPIP with a radioactivity of 51 ± 5 MBq in 0.2 mL saline. PET dynamic images were reconstructed by filtered back projection using Hanning’s filter with a Nyquist cutoff of 0.5 cycle/pixel. Reconstructed PET images were summed using analysis software (ASIPro VM™, Analysis Tools and System Setup/Diagnostics Tool, Siemens Medical Solutions, Knoxville, TN, USA). Volumes of interest were drawn on the striatum, thalamus, pons/medulla, and cerebellum referring to rat brain magnetic resonance imaging template, and the time-activity curves (TACs) in each region were characterized. Brain uptake of radioactivity was decay-corrected to the injection time and was expressed as the standardized uptake value (SUV), which was normalized to the injected radioactivity and body weight. SUV was calculated according to the following formula: SUV = (radioactivity per milliliter tissue / injected radioactivity) × gram body weight.

For the blocking study, a SD rat (*n* = 3, male, 260 to 280 g; 8 weeks old) was injected with unlabeled MMPIP (1 mg/kg) 30 s prior to the injection of [^11^C]MMPIP (51 ± 5 MBq, 0.5 nmol). The PET procedure and data analysis were performed as described above.

### Metabolite analysis in rat plasma and brain

[^11^C]MMPIP (37 to 111 MBq, 0.3 to 1.0 nmol in 0.1 to 0.3 mL saline) was injected into SD rats (male, 240 to 270 g; 8 weeks old) via their tail vein. Then, the rats that were anesthetized with 5% (*v*/*v*) isoflurane were sacrificed by cervical dislocation at 5, 15, 30, and 60 min following a bolus injection. Blood (0.5 to 1.0 mL) and whole brain samples were quickly removed after sacrifice. The blood samples were centrifuged with AT-2724 M rotor (KUBOTA, Tokyo, Japan) at 15,000 rpm for 2 min at 4°C to separate the plasma, 0.25 mL of which was added to a test tube containing CH_3_CN (0.5 mL). The tube was then vortexed for 15 s and centrifuged using the Minispin plus (Eppendorf, Hamburg, Germany) at 14,500 rpm for 2 min for deproteinization, and the supernatant was collected. The brain samples were homogenized together in ice-cooled CH_3_CN/H_2_O (1:1, 1.0 mL) solution using the Silent Crusher S (Heidolph Instruments, Schwabach, Germany). The homogenate was centrifuged for 2 min at 4°C and 0.5 mL of the supernatant was added to a test tube containing CH_3_CN (0.5 mL). The tube was then vortexed for 15 s and centrifuged for 2 min for deproteinization, and the supernatant was collected. An aliquot of the supernatant (0.1 to 0.3 mL) prepared from the plasma or brain homogenate was analyzed by HPLC with a highly sensitive detector for radioactivity [18] using a Capcell Pak UG-80 C_18_ column (4.6 mm ID × 250 mm) and CH_3_CN/H_2_O (1:1) at 1.5 mL/min. The percentage of unchanged [^11^C]MMPIP to total radioactivity (corrected for decay) on the HPLC charts was calculated as the percentage of the parent fraction = (peak area for [^11^C]MMPIP / total peak areas) × 100.

TACs of intact [^11^C]MMPIP in the brain were generated from TACs of total radioactivity corrected by metabolite fraction with three exponential fitting using PMOD software (version 3.2, PMOD Technologies, Zurich, Switzerland). TACs of metabolites were obtained by subtracting the intact from the total radioactivity.

## Results

### Chemistry

**Figure 2:**
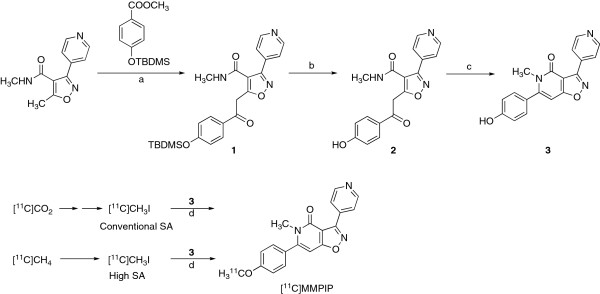
**Chemical synthesis and radiosynthesis.** (a) Lithium bis(trimethysilyl)amide in hexane, THF, −40°C, 30 min then room temperature, overnight. (b) Bu_4_NF, THF, room temperature, overnight. (c) *p*-Toluenesulfonic acid monohydrate, 1,4-dioxane, reflux, 12 h. (d) DMF, NaOH, 80°C, 3 min.

The phenol precursor **3** for radiosynthesis was synthesized according to the reaction sequences as shown in Figure 2. Reaction of methoxyisoxazole with lithium bis(trimethysilyl)amide, followed by treatment with 4-substituted benzoate, gave 5-(2-phenyl-2-oxoethyl)isoxazole **1**. After cleavage of the protecting group in **1**, the obtained **2** was treated with *p*-toluenesulfonic acid to give precursor **3**.

[^11^C]MMPIP with two different SA levels was synthesized by *O*-[^11^C]methylation of precursor **3** with [^11^C]CH_3_I in DMF at 80°C for 3 min (Figure 2). As for the conventional SA, [^11^C]CH_3_I was produced by reduction of the cyclotron-produced [^11^C]CO_2_ with lithium aluminum hydride, followed by treatment with HI. HPLC separation for the reaction mixture gave [^11^C]MMPIP with 590 ± 80 MBq (*n* = 5) as an injectable solution of sterile normal saline at the EOS, starting from [^11^C]CO_2_ of 19 ± 4 GBq, which was produced by 12- to 15-min proton (14.2 MeV on target) bombardment using a beam current of 15 μA. The decay-corrected radiochemical yield and synthesis time was 7.3% ± 1.2% and 25 min from the end of bombardment (EOB). At EOS, the radiochemical purity and SA was 98.3% ± 0.4% and 58 ± 25 GBq/μmol (*n* = 5). The radiochemical purity of [^11^C]MMPIP remained at 96.0% ± 0.7% (*n* = 3) after maintaining this product at room temperature over 120 min.

As for the high SA, [^11^C]CH_3_I was produced by iodination of the cyclotron-produced [^11^C]CH_4_. At EOS, 800 ± 170 MBq (*n* = 5) of [^11^C]MMPIP was obtained starting from 39 ± 2 GBq of [^11^C]CH_4_, which was produced by 30-min proton bombardment using a beam current of 20 μA. The decay-corrected radiochemical yield and synthesis time was 5.1% ± 0.7% and 27 min from EOB. At EOS, the radiochemical purity and SA was 97.8% ± 0.6% and 3,800 ± 625 GBq/μmol (*n* = 5). [^11^C]MMPIP of high SA also showed stability (>95.0% radiochemical purity, *n* = 3) for radiolysis after maintaining this product over 120 min.

### Measurement of Log *D* and computation of cLog *D*

The measured Log *D* value of [^11^C]MMPIP was 3.17 ± 0.02 (*n* = 3). The computed value of cLog *D* for MMPIP was 2.95.

### *In vitro* autoradiography

**Figure 3:**
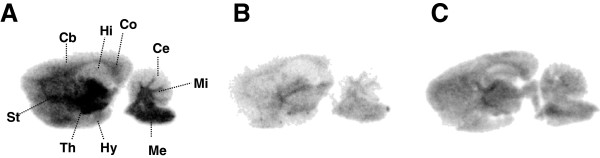
***In vitro *autoradiography using rat brain sections.** Slides were incubated with [^11^C]MMPIP including the vehicle **(A)**, 10 μM of AMN082 **(B)**, or 10 μM of unlabeled MMPIP **(C)**. Cb, cerebral cortex; Ce, cerebellum; Co, corpus callosum; Hi, hippocampus; Hy, hypothalamus; Me, medulla oblongata; Mi, middle peduncle; Th, thalamus; St, striatum.

Figure 3A,B,C shows representative *in vitro* autoradiograms using [^11^C]MMPIP co-incubated with vehicle, 10 μM of AMN082, or 10 μM of unlabeled MMPIP, respectively. In the control section, strong radioactive signals were observed in the thalamus and medulla oblongata, followed by the striatum, middle peduncle, and cerebral cortex (Figure 3A). By co-incubation with excess AMN082 (Figure 3B) or unlabeled MMPIP (Figure 3C), the radioactive signals in the brain section were homogeneously decreased, compared to the signals in the control section. The decrease rate of radioactive signals in each brain region was 34% to 53% for AMN082 treatment and 41% to 60% for unlabeled MMPIP treatment, respectively.

### Small-animal PET

**Figure 4:**
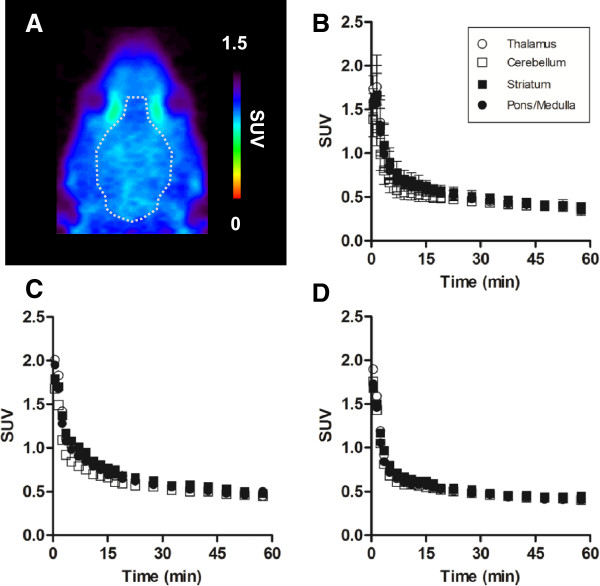
**PET studies with [**^**11**^**C]MMPIP in the rat brain. (A)** representative PET images of [^11^C]MMPIP with conventional SA on the horizontal slice. **(B)** time-activity curves (TACs) of [^11^C]MMPIP with conventional SA (58 GBq/μmol) in the thalamus (open circles), cerebellum (open squares), cerebral cortex (open triangles), striatum (filled squares), and pons/medulla (filled circles) of baseline rats. **(C)** TACs of the blocking study with unlabeled MMPIP (1 mg/kg). **(D)** TACs of [^11^C]MMPIP with high SA (3,740 GBq/μmol).

Figure 4A shows a representative PET image of the rat brain for [^11^C]MMPIP with conventional SA. In the horizontal PET image, radioactive signals weakly spread over the whole brain. There were no significant regional differences of uptake of radioactivity in all over the brain.

Figure 4B,C shows the TACs of [^11^C]MMPIP with conventional SA in the brain regions in the baseline and blocking study with excess MMPIP. All curves in each brain region showed high initial uptake but rapid clearance. No significant difference between baseline and blockade was observed.

Figure 4D shows the TACs of [^11^C]MMPIP with high SA in the brain regions. The kinetics in each region showed a rapid clearance of radioactivity peaking at the initial phase following commencement of the PET scan, which was similar to the kinetics of the conventional [^11^C]MMPIP.

### Metabolite analysis in rat plasma and brain

Table 1 shows the percentages of unchanged [^11^C]MMPIP in the plasma and brain. The fraction corresponding to unchanged [^11^C]MMPIP (*t*_R_ = 5.8 min) in the plasma gradually decreased to 48% at 15 min and 18% at 60 min after the injection. Two major radiolabeled metabolites (*t*_R_ = 2.1 and 3.0 min) were observed on the HPLC charts. On the other hand, unchanged [^11^C]MMPIP in the brain homogenate remained at 72% of total radioactivity at 15 min after the injection and decreased with time after that. At 60 min, the percentage of intact form in the brain was close to that in the plasma. Two radiolabeled metabolites in the plasma were also detected in the brain.

**Table 1:** **Percentages of radioactivity after the injection of [**^**11**^**C]MMPIP in the plasma and brain**

**Post-injection (min)**	**Plasma**			**Brain**		
	**Intact**	**Metabolite 1**	**Metabolite 2**	**Intact**	**Metabolite 1**	**Metabolite 2**
5	81.3 ± 8.4	13.4 ± 6.0	5.3 ± 2.5	92.0 ± 2.7	1.5 ± 0.5	6.5 ± 2.5
15	48.7 ± 4.1	36.7 ± 5.8	14.6 ± 2.5	72.3 ± 3.6	8.2 ± 0.7	19.5 ± 3.0
30	30.6 ± 3.7	43.3 ± 1.2	26.1 ± 4.9	36.2 ± 2.9	23.1 ± 2.4	40.7 ± 5.2
60	18.0 ± 0.3	62.1 ± 3.9	19.9 ± 3.6	18.5 ± 3.5	47.9 ± 12.7	33.5 ± 9.2

Percentages are expressed as mean ± S.D., *n* = 3.

**Figure 5:**
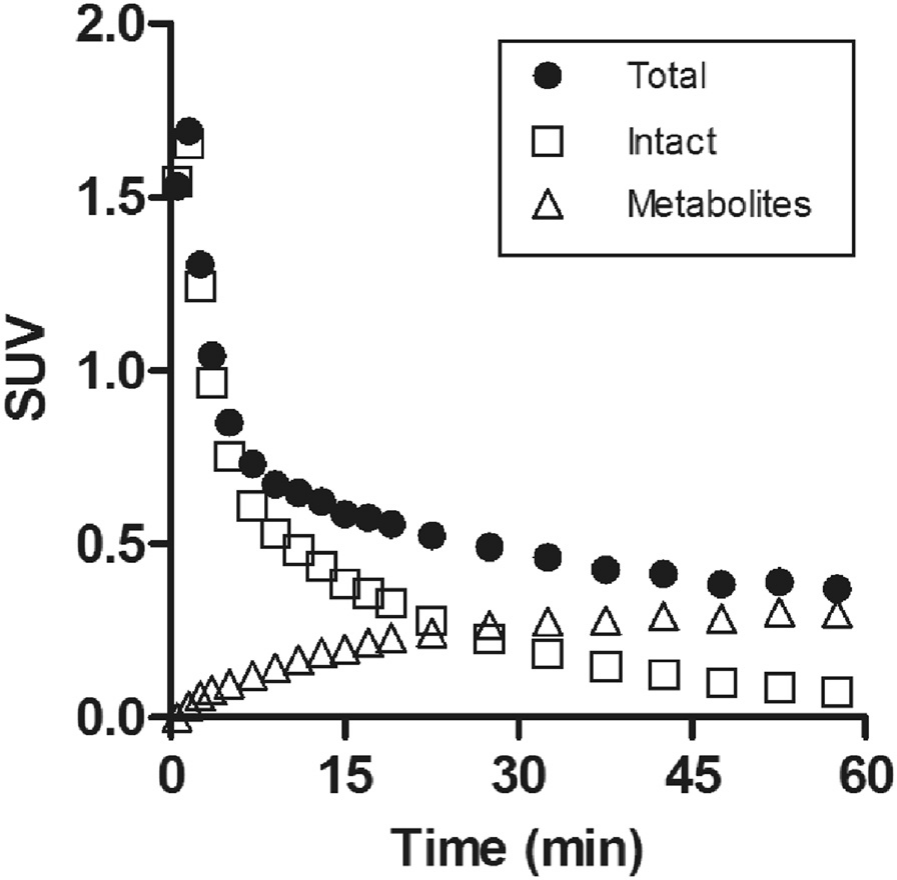
**Radioactive compositions in the rat brain.** TACs showed total radioactivity (filled circles), intact (open squares), and two metabolites (open triangles). The TACs in the intact and metabolites were corrected by a fitting curve obtained from metabolite fractions of the brain.

Figure 5 shows the TACs of total, intact, and radiolabeled metabolites in the brains. Total radioactivity decreased very slowly following a rapid decrease. In contrast, the metabolites gradually increased until 60 min after the injection.

## Discussion

Currently, mGlu7 is recognized as a targeted receptor for the development of pharmaceuticals against CNS disorders, such as epilepsy [19,20], schizophrenia [21-23], and anxiety [10,24,25]. In fact, studies on mGlu7-deficient mice directly demonstrated that mGlu7 is involved in the neurobiological mechanisms underlying the conceptually diverse phenomena of anxiety, fear extension, and spatial working memory [10]. Owing to these backgrounds, development of a PET ligand for mGlu7 has become the object of our study.

In the present study, we succeeded in the radiosynthesis of [^11^C]MMPIP (Figure 1) as a potential PET ligand for mGlu7, with two SA levels (average values 58 GBq/μmol and 3,800 GBq/μmol, respectively). To evaluate the utility of [^11^C]MMPIP for the imaging of mGlu7, we performed *in vitro* and *in vivo* studies on rat brains.

*In vitro* autoradiography with [^11^C]MMPIP was performed using rat brain sections. It has been reported that mRNA of mGlu7 was highly expressed in the hypothalamus, thalamus, cerebral cortex, striatum, and midbrain of the rat brain [7,26]. However, immunostaining with mGlu7-directed antibodies has demonstrated that the distribution of immunoactivity was particularly high in sensory areas, such as the piriform cortex, superior colliculus, dorsal cochlear nucleus, and spinal nuclei [27]. Bradley et al. explained that the difference in regional distribution between the two studies might be due to synaptic localization of mGlu7 [27]. In our autoradiogram of the control (Figure 3A), highly radioactive accumulations were detected on the thalamus and medulla oblongata, moderate accumulations were on the striatum and middle peduncle, and a low level was seen on the cerebellum. This pattern showed a comprehensive distribution of mGlu7, as also seen in the two previous reports. Moreover, by co-incubation with excess AMN082 or unlabeled MMPIP, specific binding of [^11^C]MMPIP with mGlu7 was verified in the brain regions. This finding motivated us to progress *in vivo* study.

Contrary to the *in vitro* result, *in vivo* specific binding was not observed in the rat brain using PET with [^11^C]MMPIP (Figure 4). To decrease the influence of unlabeled MMPIP that were mixed in the preparation of conventional SA on the putative specific binding, we further synthesized [^11^C]MMPIP with high SA. The mass amount of unlabeled MMPIP in the high SA preparation was 65-fold less than that in the conventional SA. PET ligands with high SA enable to successfully visualize brain receptors with small changes and to elucidate slight binding sites [28-30]. However, in the present study, there was no difference in kinetics and the distribution pattern of uptake between the two SA levels (Figure 4B,C,D).

The reasons for this failure to visualize *in vivo* specific binding of [^11^C]MMPIP with mGlu7 can be understood by considering the following factors [31]. The first is the limiting effect via ATP-binding cassette transporters [32], such as P-glycoprotein and breast cancer resistant protein, on the blood–brain barrier (BBB) against the entrance of [^11^C]MMPIP into the brain. As shown in the TACs (Figure 4B), [^11^C]MMPIP showed an acute initial uptake into the brain, reaching a maximum SUV of 1.9 at 3 min following the injection. This quick brain penetration of [^11^C]MMPIP was probably dependent on its lipophilicity (Log *D* = 3.17), which was within the range generally considered to be suitable as a PET ligand for brain imaging [31]. This result suggested that transporters did not have a significant limiting effect on the brain penetration of this radioligand.

The second is the influence of radiolabeled metabolites of [^11^C]MMPIP in the brain. If the radiolabeled metabolites penetrate into the brain, signals of specific binding to a target receptor may be obscured by non-specific binding signals and hidden by background signal. In the present study, although unchanged [^11^C]MMPIP in the brain was retained at >70% at 15 min after the injection, the percentage of intact form decreased to a level close to that in the plasma at 60 min (Table 1). In fact, the radioactivity level of metabolites increased in the brain with time after the injection (Figure 5). These results suggested that the radiolabeled metabolites in the brain may weaken the putative specific signals of [^11^C]MMPIP for mGlu7 to some extent.

The third and perhaps most important factor is the binding affinity of [^11^C]MMPIP for mGlu7. The ratio of the density (*B*_max_) of target receptors in the brain regions of interest to the equilibrium dissociation constant (*K*_D_) of a radioligand [33] is commonly used to predict the magnitude of specific binding in a PET study with the radioligand. In the present positron-emitter radioligands, the ratio of *B*_max_/*K*_D_, also referred to as binding potential, should ideally be greater than 2 to achieve a measurable specific binding on the basis of individual experiences [34-36]. For example, the *K*_D_ value of [^11^C]raclopride, a PET ligand for dopamine D_2_ receptor, was roughly 8 nM, and *B*_max_ of D_2_ receptor in the striatum was 20 nM; the ratio of *B*_max_/*K*_D_ was about 2.5 [37].

It is known that mGlu receptors in group III are expressed widely throughout the brain at both mRNA and protein levels, and they are present at both glutamatergic and GABAergic terminals [38]. However, to our knowledge, there has been no report involving the individual density of mGlu7 to date. The density (*B*_max_) of other mGlu receptors has been reported, e.g., mGlu1, 82 nM in the cerebellum of rat brain [39]; mGlu5, roughly 120 nM in the rat brain homogenate [40]. If mGlu7 is expressed at a similar *B*_max_ to mGlu1 or mGlu5 in the rat brain, mGlu7 might be visualized using [^11^C]MMPIP, because the functional binding constant (*K*_B_) of MMPIP for mGlu7 was reported to be 30 nM [12]. However, the present results give insights such that the mGlu7 density in the rat brain may be much lower than the density of mGlu1 or mGlu5. Thus, to achieve visualization of mGlu7 in the brain with PET, the development of novel ligand candidates with at least one order higher binding affinity for this receptor than MMPIP is required.

## Conclusion

In the present study, we succeeded in achieving the radiosynthesis of [^11^C]MMPIP with two different SA levels as the first potential radioligand for mGlu7 and demonstrated its specific binding using *in vitro* autoradiography. However, visualization of mGlu7 in the rat brain using PET was not successful because of the low affinity of MMPIP for mGlu7. In addition, radiolabeled metabolites entering the brain might also weaken the signal of specific binding of [^11^C]MMPIP to some extent. In the future, it is necessary to develop new ligand candidates which have higher binding affinity to mGlu7 and a more suitable profile of metabolism than MMPIP for the PET imaging of mGlu7 in the brain.

## Competing interests

The authors declare that they have no competing interests.

## Authors’ contributions

TY participated in all experiments and drafted the manuscript. K Kumata, MF, and K Kawamura performed the chemical synthesis, radiosynthesis, and measurement of lipophilicity. JY, AH, and XL carried out the autoradiography, PET experiments, and metabolite analyses. KF, MO, and NN operated the automated machine for radiosynthesis. M-RZ designed the present study and wrote the manuscript. All authors read and approved the final manuscript.
